# Towards Employee Creativity in the Healthcare Sector: Investigating the Role of Polychronicity, Job Engagement, and Functional Flexibility

**DOI:** 10.3390/healthcare9070837

**Published:** 2021-07-01

**Authors:** Junaid Waheed, Wen Jun, Zahid Yousaf, Magdalena Radulescu, Hadi Hussain

**Affiliations:** 1 School of Economics and Finance, Xian Jiaotong University, Xian 710000, China; junaidwaheed@stu.xjtu.edu.cn (J.W.); wjun1978@163.com (W.J.); hussainhadi@stu.xjtu.edu.cn (H.H.); 2 Higher Education Department, Government College of Management Sciences, Mansehra 21300, Pakistan; 3 Department of Finance, Accounting and Economics, University of Pitesti, 110040 Pitesti, Romania; magdalena.radulescu@upit.ro

**Keywords:** healthcare sector, employee polychronicity, employee creativity, job engagement, functional flexibility, nurses

## Abstract

Given the importance of individual level creativity, this paper investigates the influence of employee polychronicity on employee creativity among nurses in the healthcare sector. The current research also tests how job engagement acts as a mediator between employees’ polychronicity and creativity. Finally, thepaper analyzes the role of functional flexibility as a moderator that enhances the influence of polychronicity on employee creativity. The current paper presents empirical research, and cross-sectional data were gathered from 457 nurses (Subordinate Staff) and 127 doctors (Supervisors) working in 37DHQ (District Head Quarters) hospitals in Pakistan. Descriptive statistics, correlation, and multiple-regression techniques were applied for analyzing the collected data. The findings proved that the nurses’ polychronic attitude increases their creativity. Findings revealed that job commitment plays a mediating role between polychronicity and employee creativity. The findings proved that functional flexibility enhances the link between polychronicity and creativity. This research has contributed to both theory and managerial practice about the interplay of polychronicity, creativity, job engagement, and functional flexibility among nurses. The management in practice should focus on employee attitude, i.e., polychronicity, for improving their creativeness.

## 1. Introduction

In the healthcare sector, the nursing profession is a critical job, and nurses have to face many challenges in their jobs [1]. In developing countries, the nursing staff is considered as low-profile staff (lower cadre/grade), and they have to perform many tasks at once [2]. The nature of their duties in hospitals is very complex, and nurses are bound to perform several tasks in parallel. Such busy occupational requirements push them towards creativity, so they can discover the newest ways to perform these tasks simultaneously; hence, polychronicity is required. Polychronicity represents each one’s preference for dealing with many tasks simultaneously [3,4,5].

Existing creativity and innovation-related deliberations have been applied to all types of organizations due to rapid advancements in technology [6]. However, limited studies are available regarding creativity in the area of the service sector and more specifically in healthcare [7]. The healthcare sector has been recognized as an important and life-saving actor during the COVID-19 pandemic [8]. Therefore, the advancement of the healthcare sector becomes crucial in order to cope with the changing environment all over the world. Creativity at an individual level becomes a critical mechanism for the development of employees working in the healthcare sector in the era of advanced technologies [7]. In this regard, nurses’ creativity has gained more attention of researchers in the field of healthcare [9]. In addition toother factors, the most influential factor that shapes nurses’ creativity is their own abilities and attitudes. Among these factors, the most important one is their polychromic attitude and level of engagement at work. However, theliterature lacks insights into employee creativity through polychronicity, job engagement, and functional flexibility. Therefore, an explicit study is required to realize the role of nurses’polychronicity on their job engagement and creativity in the workplace.Creativity represents the development of new and helpful ideas that increase the efficiency and effectiveness of different processes [10].

Previous researchers acknowledged that polychronicity positively influences the overall organizational performance, sale increases, and personal level of work performance [11]. However, the direct link between polychronicity and employee creativity has not been explored so far, specifically in developing nations such asPakistan. To overcome this research gap, this paper tries to enhance the existing knowledge by analyzing the direct impact of polychronicity on creativity. The current paper also analyzes job engagement (JE) as a mediator between the polychronicity and creativity link. JE refers to an optimistic, satisfying work-related mental condition featured by liveliness, devotion, and commitment [12], although numerous researchers have linked JE with job performance [13], job requirements, job means [12] and job satisfaction [13]. There is no single study showing the effect of polychronicity on creativity using JE as a mediator. To fill this gap in the existing knowledge on this specific topic, the current research was initiated.

Investigating the sound results and the influential role of polychronicity, this research also uses functional flexibility as a moderator between the polychronicity and creativity link. Functional flexibility represents an individual adopting the decision to perform extra-role tasks. This paper brings together the concepts of polychronicity, JE, and functional flexibility for reaching employee creativity and builds a model for the healthcare sector.

The current research aims to show that polychronicity is a significant factor of employee creativity and investigates the way that JE mediates the link between employee polychronicity and employee creativity. This paper also tested functional flexibility as a moderating factor which enhances the effect of polychronicity on employee creativity. Section 2 presents the findings of previous studies on the variablesand the development of hypotheses.The next sections present the methodology and data analysis. The last section comprises the major findings that conclude the paper, theoretical and managerial implications, and some directions for future investigation.

## 2. Literature

The notion of creativity remains a central part of focus agenda for all organizations [14]. Additionally, this shift of focus is more profound in the healthcare sector for a number of reasons [9]. Among these reasons for the emergent demand for workforce creativity in the healthcare sector are COVID-19 related issues. The COVID-19 pandemic has changed patterns of work for doctors and nurses [8]. Each day, working staff in the healthcare sector face new technologies, new equipment, and new techniques. To face these challenges, this work force must be equipped with creative ideas; therefore, the employee’s creativity is the demand of current decade, specifically for the healthcare sector [7]. The current study shows how employee creativity is initiated through polychronicity and job engagement. Polychronicity enable nurses to think of the newest methods and techniques. The polychronic behavior of nurses itself is a major antecedent of employee creativity [10]. In addition to this direct impact, the current study shows that creativity is related to one own’s commitment and effort, so polychronicity alone is not enough to prolong employee creativity in the healthcare sector, and there is the missing link of employee job engagement [15,16]. Therefore, this study investigates the mediating role of job engagement between polychronicity and employee creativity. This study also shows that employee functional flexibility speeds up the link between polychronicity and employee creativity.

### 2.1. Hypothesis Development

#### 2.1.1. Employee Polychronicity and Employee Creativity

The concept of polychronicity was firstly used to explain the preference of people to do numerous tasks simultaneously (i.e., more than one task at the same time); furthermore, employees with polychronicity believe they are good at performing several tasks at once rather than doing one task [17]. Polychronic individuals display the preference to perform many tasks simultaneously and change their focus among different tasks, instead of focusing on a single issue [18,19]. Polychronic employees perform a combination of various activities at a given time and perform work in the best way by handling related disruptions [1]. Polychronicity does not include employee behaviors to manage simultaneous tasks, but it includes employee preferences to perform multiple tasks at once [20]. Therefore, this study argues that the employee preference for engaging in multiple tasks enhances employee creativity.

Polychronic individuals place importance on performing multiple things at once. This includes task switching, where employees move among different things in a given timeframe [21]. This research argued that an employee’s polychronicity influences employee creativity by developing skills to initiate unique, new, and useful ideas to complete various tasks at once. The literature has suggested that polychronicity is studied with job resources, job demand [12], aim orientation, error orientation [22,23], job performance, organizational performance [3], media multitasking, executive functions [18], job satisfaction [14], sale growth [24], and organizational identification [20]. The literature lacks a focus on employees’ polychronic attitudes towards employee creativity.

Creativity is explained as the development of new, unique, and helpful ideas that are important for the effectiveness and efficiency of various processes [25]. It is argued that creativity is a prerequisite for new and useful ideas, products, and services. In [6], it is stated that creativity is the outcome of individuals’ creative thinking and expertise gained from education and past experiences. Creativity in employees is a process that goes beyond and above normal behaviors [26]. The literature highlights that employee creativity is associated with empowering leadership [27], transformational leadership [28,29], value addition [30], employees’ perceptions of CSR [6], innovation leadership [31], employee deviance [32], and competitive advantage [33]. This research investigates the link between polychronicity and employee creativity. Moreover, limited attention has been paid to the link between polychronicity and employee creativity.

Polychronicity allows employees to be involved in different tasks at once; therefore, workers with polychronicity try to find and explore new ways and methods to perform their tasks [18,19,20]. The efforts exerted by employees to find new ways to accomplish multiple things at one time help employees to be more creative than conventional procedures [32]. The previous researcher in [34] stated that employee polychronicity helps to start the process of creativity and innovation. Therefore, the creativity of employees mainly depends on the importance givento employees who wish to perform multiple tasks at once. Such importance given to performing several things at once is charged by polychronicity. Employee polychronicity is vital for promoting creative thinking and changing work behaviors from conventional ways [14]. Paramedic staff needs to be more polychronic, as effective time usage has been considered as an important area for better behaviors in the workplace. Time use has received much attention from researchers in polychronicity [32]. Nurses in hospitals need to be involved in multiple issues at once. Polychronicity in nurses explains how they switch and prefer to complete different issues in a specified period of time [20]. If nurses are not able to deal with many tasks simultaneously, they need to be more creative to perform several things at once [18]. Therefore, a polychronic attitude triggers employee creativity. Workload variations and job nature are very sensitive and routine matters for nurses; therefore, a polychronic attitude is important for maximum job performance. Multitasking might affect service delivery; to ensure good medical care, the adoption of creativity is important to deal with many tasks simultaneously [35]. Responsibilities for nurses, such as patient care, include medication, dressing, beddings, and record-keeping that requirethem to be more creative for better service quality. Hence, this suggests that polychronicity is a significant factorthat influences creativity.

**Hypothesis 1.**  *Employee polychronicity is positively linked to employee creativity.*

#### 2.1.2. Job Engagement Mediates Polychronicity and Creativity

The notion of job engagement was first devised by the previous researcher [36], who explained job engagement as an individual’s concentration and ability to work and put in effort within the group. Job engagement is a condition where an employee is involved in his work such that it satisfies all three levels of consciousness, that is, affective or emotional, physical, and cognitive levels [36]. Emotions cover the affective part, where the employee is satisfied with the task assigned and happily working on it. Physical consciousness includes intrinsic motivation, individuality, and control; moreover, cognition includes the involvement of employees in work. Subsequently, job engagement directs employees to put effort into their targeted goals [37]. Research findings such as [38] show that job engagement is a fine job-related mindset that consists of absorption, dedication, and strength. Strength describes the energy and spark required during work, also defined as readiness to put effort into accomplishing the assigned task. Dedication means one’s level of passion, enthusiasm, pride, and inspiration. The third level, absorption, refers to the obsession, concentration, and absorption of the work. This study posits that job engagement makes a connection between polychronicity and employee creativity. Similarly, committed nurses have more propensity to present proactive behavior and active personal ideas with high motivation, which can generate a new set of procedures for doing the entire job through an increased creativity. Seemingly few research studies determined the role of job engagement for good job performance [13], job requirements and means [12], and job satisfaction [14]. However, job engagement as a bridge between polychronicity and employee creativity has not yet been investigated; more specifically, this area is under researched in the context of nursing staff. Hence, this study argued that polychronic employees have a number of reasons to work in the existing position that enables them to work on multiple tasks at the same time. This psychological empowerment helps nurses to become attached to their jobs and try to find new ways to perform their tasks [39]. However, polychronicity creates job engagement that gives benefits to employees, and they feel more attractive and able to attain freedom. This perception of a secure future and freedom allow employees to be more creative. Job engagement helps employees to improve their ability concerning the creation of new concepts, new solutions, and unique work attitudes.

Polychronic employees have the preference of performing multiple things at one time, which helps employees to enhance their job engagement in terms of absorption, strength, and commitment. Polychronicity promotes vigor among employees by creating self-confidence that triggers employees to take risks to enhance creativity. Furthermore, polychronicity also helps employees to increase their creativity by developing abilities, dedication to their efforts, and time for the organization. Lastly, handling multiple issues generally requires a significant sacrifice of time and effort. Sacrificing their spare time and energy helps employees to motivate themselves, become involved in their work, and try to find some creative ideas to perform their tasks. Thus, job engagement (including all three dimensions: strength, commitment, and absorption) mediates the link between polychronicity and employee creativity.

Polychronicity creates a basis for increased job engagement among employees, and they show more commitment to creativity in daily behaviors and activities. Furthermore, polychronicity in employees offers a comprehensive mechanism that can strengthen their creativity through job commitment. The current paper theorizes that job commitment mediates the link between polychronicity and IWB. We argue that job engagement should act as a mediating relationship between nurses’ polychronicity and creativity, as the polychronicity of nurses can enhance the creativity of employees when they are engaged in their work. Nurses are more creative in their routine tasks when they are devotionally engaged in their work [40,41]. According to a previous study [42], job engagement is the result of polychronicity. In developing countries, nurses leave their job as they have to face many difficulties and to perform hectic job activities [43]. Job engagement increases the likelihood that they will not leave their job and will try to find creative and useful ideas to perform multiple things at the same time [44]. Therefore, we argue that job engagement links polychronic nurses with creativity. Job engagement ensures that nurses engage in their job with full attention to perform numerous tasks at once to be more creative [41]. As [45] stated, nursing is a noble profession that directly serves humans. It is further argued that without job engagement, it is not possible for every nurse to perform multiple tasks with creativity at once, as job engagement mediates the link between polychronicity and creativity. Job engagement acting as mediator between the polychronicity and creativity of nurses increases the likelihood that they will perform multiple tasks. Job engagement reduces the turnover intention in nurses, which results in job engagement linking polychronicity with creativity [41,42].Therefore, the above debate determines the next hypothesis.

**Hypothesis 2.**  *Job engagement mediates the link between polychronicity and employee creativity.*

#### 2.1.3. Functional Flexibility Moderates Polychronicity and Creativity

Employees functional flexibility refers to employees’ ability to perform multiple functions and perform responsibilities other than their routine tasks [46]. These are individual-level skills through which employees are able to adapt to changing situations according to requirements [1]. This study argued that the polychronic attitude of nurses working in hospitals causes them to prefer to perform several tasks, which improves their creativeness, and this relation will be quicker when employees have high functional flexibility. Functional flexibility asa process of changing working roles accordingly helps the polychronic nurses to act in a creative way [19]. Hence, the functional flexibility of nurses is important for improving the impact of polychronicity on creativity [47]. Functional flexibility is an important process to achieve diverse tasks, and when employees are polychronic, i.e., prefer to perform several tasks, the creativity level of employee will be higher [48]. Functional flexibility is employee’s capabilities, such as diversity of skills, and an extra-role behavior, which is important to boost the effect of polychronicity on creativity.

The previous researcher [49] suggested that individuals required polychronicity to engage in various tasks instead of focusing on a single task. Likewise, employee creativity required an improved adaptive skill, i.e., functional flexibility provides help for the employees to enact committed efforts to give their valuable energy in new idea generation and application, which supports employee creativity. Functional flexibility provides a favorable infrastructure in this context. Functional flexibility is important in this specific frame because it supplies a good groundwork.

**Hypothesis 3.**  *Functional flexibility positively moderates the link between polychronicity and employee creativity.*

**Figure 1:**
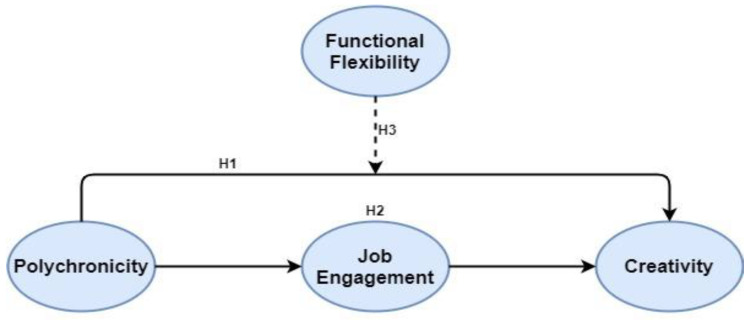
Theoretical Framework.

Theoretical framework is presented in Figure 1

## 3. Methodology

### 3.1. Data Collection

The current research applied a cross-sectional design for the empirical findings of the hypothesized model. Collection of data consists of two phases. The reasons for conducting the survey in two different phases were to reduce respondent fatigue and to minimize chances of common method variance by applying temporal separation. Before launching the full range data collection process, a pilot study was carried out on 15 doctors and 35 nurses from the said hospitals for assuring the clarity, suitability, and relevancy of the research instrument [50]. With a view to ensure proper understanding of the nature of the study vis-à-vis pertinent constructs, the questionnaire pertaining to the managers was provided in the local language (i.e., Urdu), and later on, the same was transcribed back into English. The questionnaire was checked from the experts and academics to avoid any confusion or deficiency before its distribution among respondents.

For final data collection, 37 government hospitals operating in Pakistan were selected. A list of 1700 nurses and 170 doctors was identified with the help of HR departments of these hospitals. After many sessions, the final workable responses from nurses were 457 and from doctors were 127.

#### 3.1.1. Polychronicity

Polychronicity based on 5-item scale was measured, and it was adapted from the previous study [2]. The factor analysis for polychronicity items on 1 factor results in 59.23% of the variance, and the Cronbach value was 0.861. The sample item of self-reported construct was “I prefer to do two or more activities at the same time”.

#### 3.1.2. Job Engagement

JE was rated with an 18-item scale adapted from the work of [13]. The factor analysis for job engagement was performed, and a single factor accounted for 56.43% of the variance, and Cronbach’s α value was 0.837. The sample item of self-reported construct was “I work with intensity on my job” (Physical engagement); “I am enthusiastic in my job” (Emotional engagement); “At work, my mind is focused on my job” (Cognitive engagement).

#### 3.1.3. Employee Creativity

The scale of employee creativity was rated with a 9-itemscale, i.e., adapted from previous work [51]. The factor analysis for employee creativity results in 58.23% of the variance, and Cronbach’s value was 0.828. The sample item asked from supervisors was to rate his/her subordinate: the extent to which this nurse has “Demonstrated originality in his/her work”.

#### 3.1.4. Functional Flexibility

The mediating variable functional flexibility was rated by 13 items and adaptedfrom the previous work of [1]; the sample items include: “I am broadly skilled and can carry out several tasks in this organization”, “I can quickly adapt to new situations in the organization”, “I am ready for a change of my job position of tasks within my organization”, “I am willing to devote time and energy to education in order to develop myself for a future job”, “With my knowledge and experience, I would be able to change to another job within the organization”, etc. The results charged on a single factor account for 55.14% of the variance, and Cronbach value was 0.841.

#### 3.1.5. Control Variables

The control variables include marital status, age, education, experience, and working hours of respondents.

## 4. Analysis and Results

The descriptive, correlation, and multiple-regression techniques were applied. The Harman test was conducted to examine the common method bias. The techniques from [52] were followed for confirming validity checks, and the robustness of this model was examined with Cronbach’s α.

### 4.1. Confirmatory Factor Analysis (CFA)

CFA was elaborated following the guidelines of [53], and four different models were analyzed. The results of CFA confirms that data is fit and the four-factor model has fit indices (χ^2^ = 1543.21, df = 698, *p* < 0.001, RMSEA = 0.04, GFI = 0.94, CFI = 0.93). All values are within the cutoff range and proved the model fitness [54]. CFA results are shown in Table 1.

### 4.2. Common Method Bias

The findings underline the existence of 17 factors with eigenvalues greater than 1, rather than a single factor. Moreover, these 17 factors brought 47% (approximately) of the total variance, and the first factor brought 16% of the variance. Thus, common method bias is not an issue using the Harmon’s Test approach.

### 4.3. Validity and Multicollinearity

The soundness of the constructs built in this research is not an issue, as the values of α(alpha) and composite reliability were greater than 0.80. Factor loading (FL) and values of AVE were greater than 0.60 and 0.50, confirmed convergent. Discriminant validity is also proved by comparing shared variance and average variance, i.e., AVE > shared variance.VIF (values of tolerance) were more than 0.2 and less than 10, which confirmed that multicollinearity is not a major problem in the case of this research. The figures of alpha, CR, and AVE are depicted in Table 2.

### 4.4. Correlation

The results of the correlation shows that nurses’polychronicity and nurses’ creativity are positively associated (r = 0.29). Polychronicity and job engagement have a positive association (r = 0.35). Job engagement and nurses’ creativity are also positively associated (r = 0.41). Hence, the proposed theory of this research is accepted by the results (Table 3).

### 4.5. Hypothesis Testing

Hierarchical multiple-regression analysis has been used to investigate the hypotheses. Table 4 presents the coefficient values of the regression analysis. The moderating effects are presented in Model-3, -4 and -5; functional flexibility strengthens the polychronicity and creativity link. The mediating effect of job engagement is presented in Model-6 and Model-7. The results of H1 shown in Model-6 proved that polychronicity predicts nurses’ creativity (β = 0.26 **).

The hierarchical multiple-regression technique was applied to check the mediating role of job engagement between nurses’ polychronicity and nurses’ creativity link. Model-6 confirmed the direct link between polychronicity and creativity. When job engagement is added into Model-7 to check the mediating role, the coefficients of polychronicity become nonsignificant, proving that job engagement fully mediates between the polychronicity and nurses’ creativity link (β = 0.26, *p* < 0.001 to β = 0.11, *p* > 0.05). H2 of the study is accepted, and job engagement is an outcome of polychronicity for achieving nurses’ creativity.

H3 suggested the moderating influence of functional flexibility on the polychronicity and creativity link. We conducted a hierarchical regression analysis to check the moderating role of functional flexibility on the polychronicity and creativity link.

**Figure 2:**
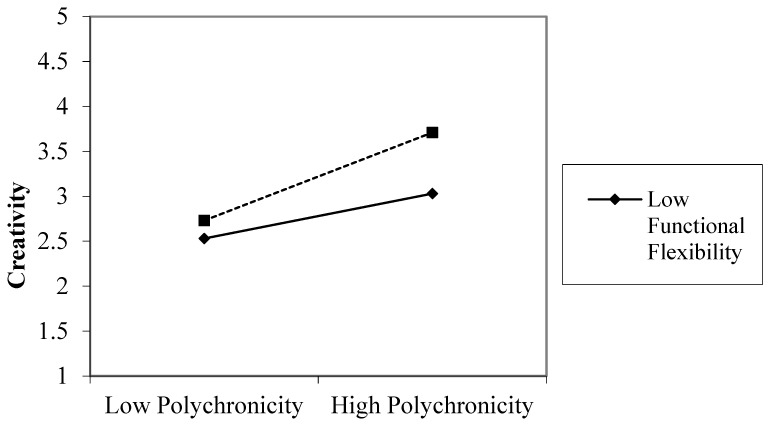
Slope analysis to test functional flexibility as moderator.

The analyses are shown in Table 4 through three models, i.e., Model-3, -4, and -5. Model-3 includes control variables, and Model-4 added both polychronicity and functional flexibility tocreativity, which are significantly associated (β = 0.28, *p* < 0.001 to β = 0.25, *p* < 0.001). Following the guidelines of [55], Model-5 presents the results of the interaction term, Poly × Functional flexibility. The results proved that the addition of functional flexibility strengthens the polychronicity and creativity link (β = 0.17, *p* < 0.001). The slope test was also shown in Figure 2, proving H3 of study.

## 5. Discussion

This study investigated employee creativity in the healthcare sector and was comprised of four hypotheses. The first hypothesis proposed that polychronicity positively influences employee creativity. The findings validated H1, and it was verified that the polychronic attitude of nurses enhances their creativity for accomplishing different simultaneous tasks. The results of the current research focus on the need for polychronicity as an urgent need to stimulate creativity among nurses. These findings highlight the underexamined segment of nurses’ and other subordinated workers’ everyday activities that can enhance their creativity through polychronicity. These findings are in line with previous researchers [2,7,34], who stated that employee polychronicity supports the process of creativity and innovation, demonstrating that employee polychronicity is vital for creative thinking and for changing work behaviors. Nurses need to be more creative to perform several things at once [9,18], so a polychronic attitude triggers employee creativity. For ensuring good medical care, creativity is important to allow nurses to deal with many tasks simultaneously [10,35].

The findings of H2 proved that JE mediates the polychronicity and employee creativity link. These results were also validated by previous studies [56] that proved that sacrificing their spare time and energy helps employees to motivate themselves and become involved in their work and to try to produces creative ideas to perform their tasks. Committed nurses have more propensity to present a proactive behavior and active personal ideas with high motivation, which can generate a new set of procedures for doing the entire job through increased creativity. This psychological empowerment helps nurses to become attached to their jobs, and they try to find new ways to perform their tasks [39]. Job engagement helps employees to improve their ability concerning the creation of new concepts and solutions and unique work attitudes. Job engagement increases the likelihood that they will not leave their job and will try to find creative and useful ideas to perform multiple things at the same time [44]. Job engagement ensures that nurses engage in their job with full attention to performing numerous tasks at once to be more creative [41,42,43,44,45]. Without job engagement it is not possible for every nurse to perform multiple tasks with creativity at once.

The last hypothesis of this research investigated the moderating role of functional flexibility, and how much it enhances the impact of polychronicity on employee creativity. The findings demonstrated that nurses’ polychronicity can improve their creativitythrough the support of functional flexibility. The organizational structures play a significant role in reaching the proposed targets. Thus, it is undeniable that for accomplishing different tasks simultaneously, the polychronic worker should search for new creative ways; however, for this, they must have the ability to adapt to new tasks. Therefore, when these subordinated workers are directly taking part in the decision-adopting process for resolving the problems they face, they will be very efficient. Thus, the effect of polychronicity on employee creativity will be quicker and more effective when employees are flexible. The findings of testing the last hypothesis of our study are validated by the previous research in [57] that highlighted that employee creativity required improved adaptive skills, i.e., functional flexibility provides help for the employees to engage in committed efforts to give their valuable energy in new idea generation and application, which supports employee creativity. Functional flexibility asa process of changing working roles accordingly helps polychronic nurses to act in creative way [17]. The functional flexibility of nurses is important for improving the impact of polychronicity on creativity and for achieving diverse tasks [38,47,48], which also proves the same boosted impact of polychronicity on creativity through functional flexibility.

### 5.1. Theoretical Implications

The current research displays many significant contributions to economic theory. First, we highlight the exciting outcome of polychronic nurses in terms of creativity. In this context, the current research built on the findings of other existing studies that investigated many other antecedents of employee creativity besides polychronicity [24]. Thus, it identified that employee creativity is a significant result of polychronicity. The existing studies connected polychronicity with organizational performance, work performance [13], sales increase [24], job requirements, job means [12], job fulfillment [14,15,16,17,18,19,20], and purpose orientation [16]. All these previous findings supported the motivation to currently analyze the polychronicity and employee creativity link. One main finding of this research was that polychronicity can be related to employee creativity. Another important result of this study is that, besides analyzing the direct link between polychronicity and employee, we have investigated the significant role played by JE in this relation.

The findings of the current research also validated the mediating role of JE for the polychronicity and employee creativity link and extended the work of previous researchers [12,13,14]. Moreover, this study aids in developing the notion of nurses’ polychronicity and enlarges the goal of polychronicity in the existing knowledge. This research builds up a wide and solid model to present the results of polychronicity by strengthening its goal. The research also investigates the link between polychronic nurses and their employee creativity to examine the motivations that determine whether these nurses are involved and committed to their jobs. Based on the empirical results and from the live experience of collecting data, we can firmly state that JE displays a mediation role for the polychronicity and employee creativity link, and this represents a significant contribution to the existing theory.

This research has also proven that the influence of polychronicity on employee creativity is even more significant when functional flexibility increases. Our results show how a decentralized organizational frame positively moderates the polychronicity and employee creativity link. Instead of underlying the most important results of functional flexibility for organizational performance, as was shown in other previous studies, this study brings significant findings for the existing knowledge by proving that functional flexibility represents a significant moderator which enhances the positive influence of polychronicity on employee creativity. It is generally accepted that the polychronic employees that aim to accomplish many tasks at the same time [5] produced creative ideas while performing many tasks at the same time when they acted in flexible and decentralized frames that permitted them to freely gain, utilize, and exchange the necessary information.

### 5.2. Practical Implications

The medical staff, especially the nurses, accomplish many assignments at the same time in their daily routine. Therefore, they need to look for creativity to accomplish these tasks simultaneously. Creativity which supports the improvement of services and organizational and administrative processes is useful. The findings of the current study also contribute to the practical management of the entire medical staff.

First, supervisors/doctors need to look closer at the subordinated staff that performmany tasks simultaneously and encourage them to enhance their creativity. They should put more emphasis on the polychronic ability of nurses to facilitate the improvement of their creativity. Such creativity of the nurses generated by their multiple job tasks will be very useful for both supervisors and nurses because conducting different activities simultaneously will lead them to find the newest ideas [58].

Secondly, the supervisors have to consider and stress the importance of JE and use it in order to enhance nurses’ creativity. The current study indicates that polychronicity can present an indirect influence on employees’ creativity through JE. Thus, the supervisors should motivate their subordinated workers more, so that they can be more involved and more committed to their jobs. These goals can be achieved by improving their interpersonal relations, team building, fair and tempting job packages, bonuses, creating emotional and mental bonds with their jobs, and strengthening their creativeness.

Finally, supervisors should fully support decentralized frames and stimulate relevant and beneficial feedback from their subordinated workers. Once all these ties, values, and efforts are settled, this will increase the positive influence of nurses’ polychronicity on their job creativity. This research can be used to show the specific ways in which supervisors can remove the inconsistencies of polychronic nurses, so they can become more creative by adding functional flexibility.

### 5.3. Limitations and Future Research

Although this current research presents important theoretical and practical implications, as previously mentioned, there are some limitations of this research that could represent some important directions for further research. Firstly, this study analyzed cross-sectional data, but the polychronicity and employee creativity link can be characterized as a progressive process. Thus, we can state that a longitudinal research construct would emphasize more interesting results for this causal relation. Moreover, a longitudinal research study can be used to avoid the common bias. Still, this bias does not represent a serious problem for the present study, as we have determined from the tests we currently applied.

## 6. Conclusions

In an attempt to unveil the impact of employee polychronicity on employee creativity, previous studies have demonstrated that polychronicity effects improve overall organizational performance. For the healthcare industry, the current study investigates a direct relation between polychronicity and employee creativity while also investigating an indirect relation via job engagement. In addition, the current study also employs functional flexibility as a moderator for the relationship between polychronicity and creativity. Overall, the study found that nurses with a polychronic attitude were more creative when completing multiple tasks at the same time, indicating that a polychronic attitude stimulates employee creativity [15,17]. It is concluded that job engagement mediates the polychronicity and creativity link. When nurses are engaged in many tasks, they will be more creative if they are attached to their jobs. Hence, JE is a precondition to establish nurses’ creativity. Finally, the data showed that nurses’ polychronicity can boost their creativity by allowing them to be more functionally flexible. As it is evident that the polychronic worker must find new innovative ways to do several jobs at the same time, they must take advantage of their functional flexibility level and have the ability to adapt to new activities. This research also found that when functional flexibility grows, the impact of polychronicity on employees’ creativity grows even more. The current study, in theory, illuminated the interesting results of polychronic nurses in terms of creativity, and it adds to the existing research on antecedents of employee creativity other than polychronicity. Furthermore, in light of JE as a mediating variable, a significant contribution to the existing hypothesis was made, extending the previous research work [2]. This study establishes a broad and robust model for presenting polychronicity outcomes by enhancing the purpose of nurses’ polychronicity and broadening the scope of polychronicity in existing literature. Alternatively, this study adds to the current body of research by demonstrating that functional flexibility is an important moderator in enhancing the favorable impact of polychronicity on employee creativity. In practice, nurses must complete multiple tasks at the same time as part of their regular routine. As a result, people must employ ingenuity in order to complete multiple activities at the same time. Supervisors must also pay more attention to subordinates who are working on multiple projects at once in order to provide them with an appropriate working environment in which to increase their creativity. They should place a greater emphasis on the nurses’ polychronicity abilities to help them increase their creativity. As a result, supervisors should encourage and motivate their subordinates to become more involved and devoted to their employment. These objectives can be met through increasing their interpersonal relationships, teamwork, fair and enticing compensation packages, bonuses, forming emotional and mental links with their professions, and enhancing their creativity. Finally, supervisors should fully embrace decentralized frames and encourage their subordinates to provide meaningful and helpful input. Once all of these links and efforts are established, the favorable impact of nurses’ polychronicity on their job creativity will expand. Considering the limitation of providing a causal explanation, the current study concludes that the relation between polychronicity and employee creativity can be described as a progressiveprocess.As a result, we can conclude that a long-term research design might yield more intriguing data for this causal relationship.

## Figures and Tables

**Table 1:** CFA results for model fitness.

Model	χ^2^	Df	χ^2^/df	RMESA	GFI	CFI
Hypothesized four-factor model	1543.21	698	2.2109	0.04	0.94	0.93
Three-factor model	1698.54	621	2.73517	0.12	0.69	0.65
Two-factor model	1689.14	538	3.13967	0.25	0.51	0.55
Single-factor model	1725.32	512	3.36977	0.39	0.57	0.53

**Table 2:** Results of alpha, CR, and AVE.

Variables Details	Alpha	CR	AVE
Employee Creativity	0.828	0.955	0.715
Functional flexibility	0.841	0.908	0.713
Job Engagement	0.837	0.967	0.701
Polychronicity	0.861	0.943	0.722

**Table 3:** Results of mean, SD, and correlations.

Variable		Mean	SD	1	2	3	4	5	6	7	8	9
1	Age	2.4	0.57	1.00								
2	Experience	2.5	0.61	−0.02	1.00							
3	Marital-Status	1.3	0.59	0.11	−0.03	1.00						
4	Working-Hours	2.6	0.63	−0.05	0.12	0.03	1.00					
5	Education	2.9	0.54	0.09	0.09	0.12	0.13	1.00				
6	Polychronicity	3.2	0.81	0.03	0.06	0.15	0.11	0.11	1.00			
7	Job Engagement	3.3	0.88	−0.08	−0.10	0.03	−0.04	0.12	0.35 **	1.00		
8	Creativity	3.6	0.91	0.10	0.07	0.05	0.05	0.09	0.29 **	0.41 **	1.00	
9	Functional flexibility	3.5	0.89	0.07	0.06	0.06	0.09	0.06	0.26 **	0.22 **	0.33 **	1.00

(** = *p* > 0.001).

**Table 4:** Results of hypotheses.

	Job Engagement		Creativity				
Variable	Model-1	Model-2	Model-3	Model-4	Model-5	Model-6	Model-7
Age	−0.024	−0.019	−0.015	−0.008	−0.009	−0.011	−0.014
Experience	−0.048	−0.021	−0.018	−0.011	−0.014	−0.024	−0.023
Marital status	0.013	0.014	0.022	0.015	0.017	0.023	0.018
Working hours	−0.054	−0.061	−0.051	−0.048	−0.038	−0.051	−0.042
Education	0.017	0.018	0.015	0.022	0.014	0.019	0.016
Polychronicity		0.36 **		0.28 **	0.37 **	0.26 **	0.11
Job Engagement			0.40 **				0.39 **
Functional flexibility				0.25 **	0.22 **		
Poly × Functional flexibility					0.17 **		
R^2^	0.04	0.33	0.39	0.36	0.35	0.25	0.37
∆R^2^	0.03	0.31	0.34	0.35	0.09	0.24	0.36
F	4.23 *	32.25 **	41.54 **	51.26 **	57.54 **	23.18 **	42.21 **
∆F	3.15 *	149.87 **	178.12 **	214.52 **	147.71 **	124.38 **	161.34 **

(* = *p* > 0.005, ** = *p* > 0.001).

## Data Availability

The data that support the findings of this study are not publicly available due to respondent confidentiality reasons. The data are not publicly available due to containing information that could compromise the privacy of the participants in this research.
